# Viral Hepatitis C New Microelimination Pathways Objective: Psychiatric Communities HCV Free

**DOI:** 10.3390/life12111873

**Published:** 2022-11-13

**Authors:** Vito Fiore, Andrea De Vito, Agnese Colpani, Valentina Manca, Ivana Maida, Giordano Madeddu, Sergio Babudieri

**Affiliations:** Unit of Infectious and Tropical Diseases, Department of Medicine, Surgery and Pharmacy, University of Sassari, 07100 Sassari, Italy

**Keywords:** chronic HCV hepatitis, microelimination programs, psychiatric disorders, DAAs

## Abstract

Background: People with psychiatric disorders have a high prevalence of HCV. For this reason, tailored interventions should be developed to reach this population. Methods: We performed a retrospective study on patients treated for HCV infection in psychiatric nursing homes, approached with a quick diagnosis, staging and treatment. Results: We included data on 586 people screened for HCV with quick tests. High HCV seroprevalence was found in this population (231; 39.4%). Among people who tested positive, there were high rates of active infection (220; 95.2%). Out of the 220 patients with active infection, 95.9% were male, 85.5% were Italian, median age was 43 (IQR = 35–52) years old. In the majority of cases (162; 73.6%), the risk factor was unknown. The most common genotype was 3a (98; 44.5%), and patients mostly had a low fibrosis, according with FIB-4 value (142; 64.5%). Of them, one (0.45%) categorically refused the treatment, and one (0.45%) had liver cirrhosis and advanced hepatocellular carcinoma. Overall, 218 patients underwent eligibility for DAAs. The most prescribed treatment was glecaprevir/pibrentasvir (GLE/PIB (172; 78.2%)). The others practiced sofosbuvir/velpatasvir (SOF/VEL). All patients reached the end of treatment. One (0.45%) was lost to follow up, and all the others reached the SVR12. Conclusions: The point-of-care testing and pangenotypic DAAs’ availability represent one of the most important steps for a fast diagnostic and therapeutical option. Tailored microelimination pathways for every difficult-to-reach/to-treat populations are needed. This would allow us to move more easily towards HCV elimination.

## 1. Introduction

Viral hepatitis C is a liver infection caused by the hepatitis C virus (HCV). Hepatitis C is transmitted through contact with the blood of infected people. Nowadays, the most common way of infection is caused by sharing needles or other equipment used to prepare/inject drugs. However, sexual intercourse still remains a method of transmission (e.g., men who have sex with men) [1]. Around 50% of infected patients will develop chronic hepatitis C (CHC) [2,3,4]. CHC may result in serious, and even life-threatening, health problems, such as liver cirrhosis and hepatocellular carcinoma (HCC).

As per Authority data, ~58 million people are affected by HCV, and ~1.5 million new infections occur every year. Even more alarming, it is estimated that ~3.2 million HCV infections occur in adolescents and children [5]. For this reason, HCV elimination represents one of the main targets according to WHO 2030 goals [6].

The actual availability of direct-acting antivirals (DAAs), with short schedules of treatment and rare/negligible adverse events, decreased the number of active infections, including difficult settings [7]. Furthermore, it is expected that, in the future, naïve and without cirrhosis will be the states of the majority of patients [8].

In Italy, the official knowledge on HCV epidemiological data is limited to 2012. In observance of World Hepatitis Day, the Italian Ministry of Health reported a HCV seroprevalence ranging from 2% to 8% in central and northern regions and in southern and insular areas, respectively [9]. Regarding genotypes, the most frequent is 1, followed by genotype 3, according to the most recent literature [10]. 

Regarding infectious diseases (e.g., HCV) among underserved populations, such as people with psychiatric disorders, there is a wide heterogeneity both on prevalence and healthcare provision and use. A defined and targeted approach to every subpopulation would be important to identify treatment gaps and create proper interventions. 

Recent literature showed how, in maintaining the current levels of diagnosis and treatment, about 60% of high-income countries are at least 20 years behind the schedule [8].

With about eight years remaining, the realization of new microelimination programmes is fundamental to increase patient outreach, particularly regarding hard-to-reach/to-treat populations.

People suffering from psychiatric disorders surely represent one of these target groups. As reported by the literature, up to 50% of patients with severe mental illness may have a history of substance abuse, with a high risk of blood-borne viruses (BBV) infections [9]. Furthermore, HCV positivity has been reported in up to 26% of psychiatry inpatients [11]. Moreover, the type of mental illness seems to influence HCV prevalence, varying from 0.4% to 19.6% according to the psychiatric disorder [12]. As is well known, HCV infection can also, itself, increase the mental illnesses burden. As a consequence, it appears clear how there is a bidirectional relationship between psychiatric illness and HCV. 

HCV management among patients with psychiatric disorders would surely benefit from better literature insight. However, to our knowledge, there are a lack of available data in this setting.

We report our experience with screening, staging and treatment with people suffering from psychiatric disorders.

## 2. Patients and Methods

### 2.1. Study Design

We conducted a retrospective study, collecting data from medical records of patients with psychiatric disorders included in programs of quick test, staging and treatment for HCV. 

### 2.2. Inclusion Criteria

Inclusion criteria were in accordance with the most successful models reported in literature [12,13,14,15]:

1. Age >18 years,
2. Being naive to DAAs,
3. Have been included in a protocol of point-of-care testing using rapid diagnostic tests (QuickOral Test^®^),
4. Having received a directly observed therapy (DOT),
5. Support administrators’ consent availability, in the case patient was not allowed to sign informed consent. If all these criteria were not respected, patients were excluded from the analysis.

### 2.3. Laboratory Assessments, Co-Infections and Treatment Schedules

HCV screening was performed with quick saliva tests (QuickOral Test^®^); HCV-RNA was performed with real-time HCV-RNA quantification (Siemens^®^). Data on HBV and HIV were carried out from patients’ medical records. Patients with active infection were prescribed DAAs, regardless of liver stage of fibrosis, as per national protocols [13].

### 2.4. Statistical Analysis

Data distribution was evaluated with a Kolmogorov–Smirnov test before the analysis. Qualitative variables were reported as absolute frequencies (percentage) and quantitative variables with means (standard deviations, SD) or medians (interquartile ranges, IQR), according to their parametric and non-parametric distribution. Data were elaborated as numbers on total (percentages), means ± SD and median (IQR). To evaluate the influence of clinical and demographic factors on the drug choice and awareness, chi-squared test with Yates correction or Fischers’ exact test was carried out, when appropriate. Logistic regression was carried out to evaluate clinical and demographic relationship with serostatus. All statistical computations were carried out with the STATA v.16 software (StatsCorp, Prosper, TX, USA).

### 2.5. Ethical Issues

We conducted this study in accordance with the Helsinki Declaration. All patients’ data were collected from routine clinical practice, fully anonymized and retrospectively analyzed. For this type of study, neither formal consent nor Ethical Committee approval are required, according to the current National Law from the Italian Medicines Agency and the Italian Data Protection Authority [16].

## 3. Results

### 3.1. Patients’ Demographics

Overall, 586 patients were tested with quick methods. Of them, 550 (93.8%) were male, 515 (87.8%) were Italian and the median age was 47 (IQR = 41–54) years. In 90.1% of cases, the risk factor was unknown. Out of the 586 included patients, 231 (39.4%) tested positive in the HCV screening. Of them, 220 (95.2%) had an active infection (positive HCV-RNA). The patients’ general characteristics have been reported in Table 1. 

### 3.2. HCV Screening Positive Patients

HCV seroprevalence was 47.9%, 49.5%, 53.7%, 55.3% and 8.6% among people with mixed pattern, major depression, schizophrenia, bipolar disorder and schizoaffective disorder, respectively. Among them, the majority had active infection (220; 95.2%). At the logistic regression, being elderly was associated with a lower risk of HCV screening positivity, as well as having a history of alcohol abuse. Regarding psychiatric disorders, people with bipolar syndrome, schizophrenia, depression and mixed patterns had a higher risk of HCV positivity compared to people with schizoaffective disorder. The logistic regression has been reported in Table 2.

When considering patients who tested negative for HCV-RNA (11; 5%), three of them (27.3%) where previously treated with DAAs, while the others (8; 72.7%) were unaware of their serological status. Probably, they had a spontaneous viral clearance.

### 3.3. Patients with active infection

Out of the 220 patients who tested positive for HCV-RNA, 211 (95.9%) were male, 188 (85.5%) were Italian, and the median age was 43 (IQR = 35–52) years. Regarding the risk factor, in the most part of cases (162; 73.6%) it was unknown; 56 (25.5%) were people who inject drugs (PWIDs). Among PWIDs, 13 (23.2%) were on opioid substitution therapy (OST). The most common disease (79; 35.9%) was a mixed pattern (mood plus personality disorder), followed by major depression (51; 23.2%). Patients’ demographics and risk factors have been reported in Table 3. 

### 3.4. Patients’ Clinical Features

Out of the 220 patients included in our study, 15 (6.8%) were people living with HIV (PLWH), and all of them practiced antiretrovirals (ARVs); 10 (4.5%) had HBsAg positivity/occult B infection.

The most common genotype was 3a (98; 44.5%), and the majority of patients had a low fibrosis, according to FIB-4 score (142; 64.5%). Patients with a FIB-4 score >3.25 and/or low platelets also underwent liver echography before DAAs prescription, to exclude HCC. The main clinical features of patients included in our survey have been reported in Table 4.

### 3.5. Previous Awareness

When analyzing awareness, age was strongly influential, with higher unawareness among people aged <55 years (unaware under 55 vs. >55 = 56 (31.6%) vs. 41 (95.3%); *p* < 0.00001). Sex was also influential, with male sex associated with higher awareness (male vs. female = 1 (11.1%) vs. 115 (54.5%); *p* = 0.0143). Interestingly, there was no difference in awareness between PWIDs and non-PWIDs (PWIDs vs. non-PWIDs = 26 (46.4%) vs. 71 (43.3%); *p* = 0.68). Instead, PLWH had higher awareness when compared with the other patients (PLWH vs. no-PLWH = 15 (100%) vs. 82 (40%); *p* < 0.00001).

### 3.6. Antiviral Prescription, Treatment and Outcomes

**Figure 1:**
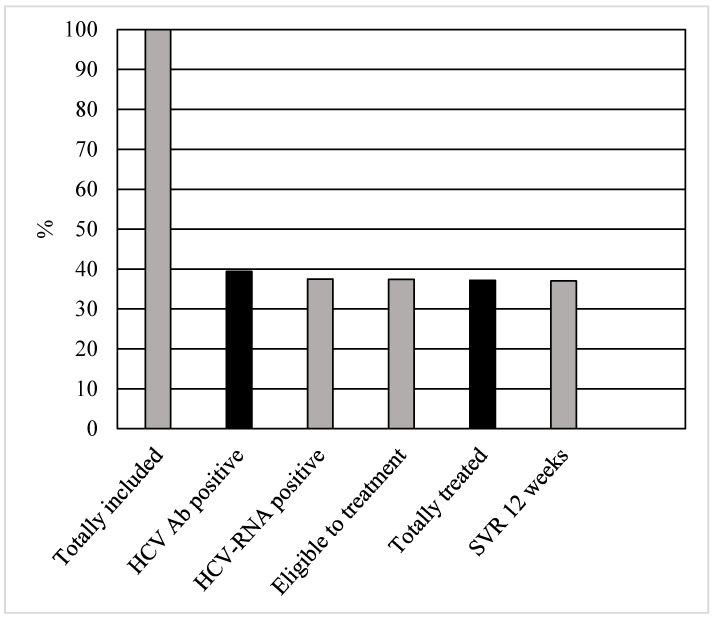
Cascade of care in 220 patients with psychiatric disorders with HCV infection.

All patients immediately underwent phlebotomy to collect HCV-RNA, genotype and exams for FIB-4 value, after performing the rapid HCV saliva test. Staging was within one week from the screening. The treatment was started within eight (IQR = 6–11) days after prescription. Out of the 220 patients, 1 (0.45%) categorically refused the treatment even if they accepted screening, staging and prescription. One patient (0.45%) had liver cirrhosis, and advanced HCC was found during echography. He underwent sorafenib for treatment. Regarding the remaining patients, all of them (218; 99%) underwent eligibility for DAAs treatment according to their drug–drug interactions. The most prescribed treatment was glecaprevir/pibrentasvir (GLE/PIB (172; 78.2%)). The other patients practiced sofosbuvir/velpatasvir (SOF/VEL). All patients reached the end of treatment. However, one (0.45%) was re-taken in charge by his family and never accompanied to controls. The overall SVR12 rate was 99.5%. The patients’ cascade of care is reported in Figure 1.

### 3.7. Age and Genotype Sub-Analysis

When performing the Chi-squared test with Yates correction to evaluate the relationship between genotype 3a and treatment prescription, this was not influential on the drug choice by the clinician (SOF/VEL vs. GLE/PIB = 78 vs. 19 (*p* = 0.72)).

Instead, patients >55 years old were more likely to undergo SOF/VEL treatment (SOF/VEL vs. GLE/PIB = 39 vs. 3 (*p* < 0.00001)). Furthermore, fibrosis influenced the choice, preferring GLE/PIB on SOF/VEL in cases of lower FIB-4 value (SOF/VEL vs. GLE/PIB= 121 vs. 20 (*p* < 0.0013)).

## 4. Discussion

Among patients with psychiatric disorders undergoing quick testing, staging treatment and DOT, we found high testing acceptance, treatment coverture and SVR12 rates (>97%). This is concordant with the available literature on difficult-to-reach/to-treat populations [14,15,16,17,18,19,20,21]. 

Most of our population was male, and this is in accordance with the literature describing underserved populations. In fact, it seems women are harder to reach in difficult settings [17].

Among the analyzed population, we found a high HCV seroprevalence (~40%). This is in accordance with previous data on HCV spread among people with psychiatric disorders. As is well known, every underserved population is plagued by different health risks. The studied group is not an exception. In 2017, Bregenzer et al. published a study on HCV prevalence among people with severe mental illness [12]. It was showed how from 30 to 50% of these patients had a history of substances abuse, underlining the high risk of BBV infections in this population [9]. 

When looking at other groups always considered to be at high risk for HCV infection and transmission, such as incarcerated people, previous studies showed a HCV seroprevalence up to 38% [22,23]. In 2021, Fiore et al. published a study showing how the prevalence of HCV among people in Italian correctional facilities was 10.4%. As an insight, PWIDs had a seroprevalence of 28%, and non-PWIDs had a seroprevalence of 5.2% [16]. Substantially, the availability of DAAs and the creation of a micro-elimination specific model reduced by three times the HCV epidemiology in prison settings.

In the same year, Girardin et al. published an article on HCV screening among psychiatry inpatients. In this case, the seroprevalence among PWIDs and non-PWIDs was of 10.8% and 25.7%, respectively [14]. Substantially, the global prevalence was ~16%: even higher than incarcerated people nowadays. Our data are in line with the literature, showing a high burden of HCV infection among people with psychiatric disorders. In the majority of cases, liver fibrosis was low. This datum is concordant with the available literature, which predicts that 75% of the future patients will be naive and without advanced disease [3]. Despite the fact that, in the most part of cases, the risk factor was unknown, we can speculate a high burden of drug abuse, given the high rates of genotype 3a we found.

Interestingly, the most common pangenotypic DAAs seemed to be influenced by age and liver fibrosis in their prescription. Instead, no influence of genotype was found. This may be related to the number of comorbidities, obviously higher among elderly, for the increase in drug–drug interactions when using protease inhibitors. 

The analysis on awareness showed interesting data. Age >55 years was influential on patients’ awareness. This was probably related to the fact that the elderly have more tests offered, given the higher possibility of health problems and more admissions to hospitals. Moreover, the majority of patients did not report particular risk factors, and the HCV positivity was less than expected. Sex seemed to be an influence, as well. However, the female population was small and needs to be confirmed from further studies. PLWH had all awareness on their serological status. This is an expected result, given these patients have a close medical follow-up for antivirals. An unexpected result was carried out from PWIDs. In fact, there was no difference between PWIDs and non-PWIDs in awareness. This is divergent from available data in literature [16]. In fact, published papers highlighted how HCV screening is offered among PWIDs in no more than 60%–70% of cases, even in territorial services [24].

Interestingly, the psychiatric disorder seems to also influence the infection.

A recent review of Gutiérrez-Rojas et al. showed a stratification of HCV prevalence among patients with different disorders. Patients with schizoaffective disorder, schizophrenia, bipolar disorder, major depression and major depression had a HCV seroprevalence of 0.4%, 2.1–16.5%, 2.6–15.5%, 25% and 17.4–19.6%, respectively [15]. Accordingly, we found a lower disease burden among people with schizoaffective disorder (~8%) than among the other groups (up to 55.3%), confirmed also by the logistic regression. However, it is difficult to compare these data, given the retrospective nature of the study. A prospective cohort with an ‘ad hoc’ sample size calculation would be needed.

As is well known, DOT is an extraordinary strategy to increase compliance in difficult settings. In our case, no patients had unplanned interruptions after the treatment’s start. 

No barriers to treatment with DAAs have been clearly highlighted by the literature. However, studies underline how the high cost of DAAs, associated with doubts about being able to reach the EOT in some populations, may sometimes be a driver for the treatments’ initiation by physicians [25,26,27]. The use of point of care testing (POCT) is an extraordinary occasion for overcoming every barrier.

## 5. Conclusions

Psychiatric patients still remain a world to explore for hepatologists. In fact, a high seroprevalence of HCV was found in our study (>39%), with high active infection rates (~95%).

The POCT and pangenotypic DAAs’ availability represent one of the most important steps for quick diagnostic and therapeutic options [28]. This would make it easier to move towards HCV elimination. However, specific microelimination pathways should be created for every difficult-to-reach/to-treat population, also reducing the possibility of advanced liver disease development and extending benefits to the whole community. DOT represents an added value when coming to patients who may have low compliance.

## 6. Limitations of the Study

Some limitations should be reported regarding this study. First of all, this is a retrospective analysis. All patients included underwent a fast-track screening, staging and DAAs prescription. A comparison with patients following a standard schedule (phlebotomy from the screening, fibroscan, etc.) would allow us to increase the study strength. Furthermore, we did not collect data on all patient’s medications and comorbidities. This would better show the influence of other factors probably influencing DAAs’ choice by the clinicians.

## Figures and Tables

**Table 1:** General characteristics of 586 psychiatric patients tested with HCV quick tests.

Variable	Result (n = 586)
Age, median (IQR)	47 (IQR = 41–54)
Male sex, n (%)	550 (93.8)
Italian nationality, n (%)	515 (87.8)
Risk factor, n (%)	
Unknown	528 (90.1)
PWIDs	56 (9.5)
MSM	1 (0.2)
Tatoo	1 (0.2)
Alcohol abuse history, n (%)	143 (24.4)
Psychiatric disorder, n (%)	
Mixed pattern	165 (28.1)
Major depression	103 (17.6)
Schizophrenia	80 (13.7)
Bipolar disorder	56 (9.5)
Schizoaffective disorder	182 (31.1)
HCV screening positive	231 (39.4)
HCV-RNA positive	220 (37.5)

PWIDs: people who inject drugs; MSM: men who have sex with men.

**Table 2:** Logistic regression analysis to assess the relationship between sociodemographic, psychiatric disorders and HCV antibody positivity in 586 people.

Variables	Univariate Analysis OR (95%CI)	*p*-Value	Multivariate Analysis	*p*-Value
Age	0.95 (0.94–0.97	<0.0001	0.97 (0.95–0.99)	0.004
Bipolar syndrome	2.22 (1.27–3.87)	0.005	8.29 (4.0–17.20)	<0.001
Schizofrenia	1.96 (1.22–3.16)	0.005	9.42 (4.81–18.46)	<0.001
Schizoaffective disorder	0.12 (0.07–0.20)	<0.001	Ref.	
Depression	1.73 (1.13–2.66)	0.012	8.92 (4.72–16.87)	<0.001
Mixed pattern	2.02 (1.40–2.92)	<0.001	7.21 (4.14–12.53)	<0.001
Alcohol abuse	0.72 (0.48–1.06)	0.100	0.50 (0.32–0.78)	0.002
Male gender	1.51 (0.73–3.14)	0.264	1.99 (0.89–4.44)	0.093

**Table 3:** General characteristics of 220 psychiatric patients who tested positive to HCV-RNA.

Variable	Result (n = 220)
Age, median (IQR)	43 (35–52)
Male sex, n (%)	211 (95.9)
Nationality, n (%)	
Italy	188 (85.5)
Romania	17 (7.7)
Albania	13 (5.9)
England	1 (0.45)
Belgium	1 (0.45)
Risk factor, n (%)	
Unknown	162 (73.6)
PWIDs	56 (25.5)
MSM	1 (0.45)
Tattoo	1 (0.45)
Alcohol abuse history, n (%)	48 (21.8)
Psychiatric disorder, n (%)	
Mixed pattern	79 (35.9)
Major depression	51 (23.2)
Schizophrenia	43 (19.5)
Bipolar disorder	31 (14.1)
Schizoaffective disorder	16 (7.3)

PWIDs: people who inject drugs; MSM: men who have sex with men.

**Table 4:** Clinical features of 220 psychiatric patients with HCV infection included in our study.

Variable	Result (n = 220)
HIV, n (%)	15 (6.8)
HBV, n (%)	
HbsAg	9 (4.1)
OBI	1 (0.45)
HCV-RNA, median (IQR)	456,500 (125,750–955,400)
Genotype, n (%)	
1a	83 (37.7)
1b	16 (7.3)
3a	98 (44.5)
4	23 (10.5)
FIB-4 score, n (%)	
Low (<1.45)	142 (64.6)
Intermediate (1.45–3.25)	59 (26.8)
Advanced (>3.25)	19 (8.6)

OBI: occult B infection.

## Data Availability

All data are available in the present manuscript.

## References

[1] Tohme R.A., Holmberg S.D. (2010). Is sexual contact a major mode of hepatitis C virus transmission?. Hepatology.

[2] Zaltron S., Spinetti A., Biasi L., Baiguera C., Castelli F. (2012). Chronic HCV infection: Epidemiological and clinical relevance. BMC Infect. Dis..

[3] Thomson E.C., Fleming V.M., Main J., Klenerman P., Weber J., Eliahoo J., Smith J., McClure M.O., Karayiannis P. (2011). Predicting spontaneous clearance of acute hepatitis C virus in a large cohort of HIV-1-infected men. Gut.

[4] Santantonio T., Wiegand J., Gerlach J.T. (2008). Acute hepatitis C: Current status and remaining challenges. J. Hepatol..

[5] WHO (2022). Hepatitis C factsheets. Switzerland..

[6] WHO (2016). Combating Hepatitis B and C to Reach Elimination by 2030 Geneva, Switzerland. https://apps.who.int/iris/handle/10665/206453.

[7] Fiore V., Ranieri R., Dell’Isola S., Pontali E., Barbarini G., Prestileo T., Marri D., Starnini G., Sotgiu G., Madeddu G. (2021). Feasibility and efficacy of 8 week Glecaprevir/Pibrentasvir to treat incarcerated viraemic HCV patients: A case-control study. Liver Int..

[8] Polaris Observatory Data. http://polarisobservatory.org/polaris/graphs.htm.

[9] Marascio N., Liberto M., Barreca G., Zicca E., Quirino A., Lamberti A., Bianco G., Matera G., Surace L., Berardelli G. (2014). Update on epidemiology of HCV in Italy: Focus on the Calabria Region. BMC Infect. Dis..

[10] Zago D., Pozzetto I., Pacenti M., Brancaccio G., Ragolia S., Basso M., Parisi S.G. (2022). Circulating Genotypes of Hepatitis C Virus in Italian Patients before and after the Application of Wider Access Criteria to HCV Treatment. Open Microbiol. J..

[11] Gamkrelidze I., Pawlotsky J.M., Lazarus J.V., Feld J.J., Zeuzem S., Bao Y., Dos Santos A.G.P., Sanchez Gonzalez Y., Razavi H. (2021). Progress towards hepatitis C virus elimination in high-income countries: An updated analysis. Liver Int..

[12] Bregenzer A., Conen A., Knuchel J., Friedl A., Eigenmann F., Näf M., Ackle P., Roth M., Fux C.A. (2017). Management of hepatitis C in decentralised versus centralised drug substitution programmes and minimally invasive point-of-care tests to close gaps in the HCV cascade. Swiss Med. Wkly..

[13] AISF (2019). Documento di Indirizzo dell’Associazione Italiana per lo Studio del Fegato per l’Uso Razionale dei Farmaci Anti-HCV Disponibili in Italia. https://www.webaisf.org/wp-content/uploads/2019/01/documento_hcv_200618.pdf.

[14] Girardin F., Painter C., Hearmon N., Eddowes L., Kaiser S., Negro F., Vernaz N. (2021). Hepatitis C prevalences in the psychiatric setting: Cost-effectiveness of scaling-up screening and direct-acting antiviral therapy. JHEP Rep..

[15] Gutiérrez-Rojas L., de la Gándara Martín J.J., García Buey L., Uriz Otano J.I., Mena Á., Roncero C. (2022). Patients with severe mental illness and hepatitis C virus infection benefit from new pangenotypic direct-acting antivirals: Results of a literature review. Gastroenterol. Hepatol..

[16] Fiore V., De Matteis G., Ranieri R., Saderi L., Pontali E., Muredda A., Ialungo A.M., Caruso R., Madeddu G., Sotgiu G. (2021). HCV testing and treatment initiation in an Italian prison setting: A step-by-step model to micro-eliminate hepatitis C. Int. J. Drug Policy.

[17] Fiore V., Rastrelli E., Madeddu G., Ranieri R., De Vito A., Giuliani R., Di Mizio G., Bolcato M., De Matteis G., Ialungo A.M. (2022). HCV spread among female incarcerated population and treatment pathways to viral elimination in Italian prison settings: Clinical perspectives and medico legal aspects. BMC Infect Dis..

[18] Schmidbauer C., Schwarz M., Schütz A., Schubert R., Schwanke C., Gutic E., Pirker R., Lang T., Reiberger T., Haltmayer H. (2021). Directly observed therapy at opioid substitution facilities using sofosbuvir/velpatasvir results in excellent SVR12 rates in PWIDs at high risk for non-adherence to DAA therapy. PLoS ONE.

[19] Schmidbauer C., Schubert R., Schütz A., Schwanke C., Luhn J., Gutic E., Pirker R., Lang T., Reiberger T., Haltmayer H. (2020). Directly observed therapy for HCV with glecaprevir/pibrentasvir alongside opioid substitution in people who inject drugs-First real world data from Austria. PLoS ONE.

[20] General Authorisation to Process Personal Data for Scientific Research Purposes—1 March 2012 [1884019]. https://www.garanteprivacy.it/home/docweb/-/docweb-display/docweb/1884019.

[21] Pontali E., Fiore V., Ialungo A.M., Ranieri R., Mollaretti O., Barbarini G., Marri D., Prestileo T., Dell’Isola S., Rastrelli E. (2018). Treatment with direct-acting antivirals in a multicenter cohort of HCV-infected inmates in Italy. Int. J. Drug Policy.

[22] Babudieri S., Longo B., Sarmati L., Starnini G., Dori L., Suligoi B., Carbonara S., Monarca R., Quercia G., Florenzano G. (2005). Correlates of HIV, HBV, and HCV infections in a prison inmate population: Results from a multicentre study in Italy. J. Med. Virol..

[23] Sabbatani S., Giuliani R., Fulgaro C., Paolillo P., Baldi E., Chiodo F. (2004). HIVAb, HCVAb and HBsAg seroprevalence among inmates of the prison of Bologna and the effect of counselling on the compliance of proposed tests. Epidemiol. Prev..

[24] Bachhuber M.A., Cunningham C.O. (2013). Changes in testing for human immunodefi- ciency virus, sexually transmitted infections, and hepatitis C virus in opioid treatment programs. JAMA.

[25] Litwin A.H., Harris K.A., Nahvi S., Zamor P.J., Soloway I.J., Tenore P.L., Kaswan D., Gourevitch M.N., Arnsten J.H. (2009). Successful treatment of chronic hepatitis C with pegylated interferon in combination with ribavirin in a methadone maintenance treatment program. J. Subst. Abuse Treat..

[26] Vroling H., Oordt-Speets A.M., Madeddu G., Babudieri S., Monarca M., O’Moore E., Vonk Noordegraaf-Schouten M., Wolff H., Montanari M., Hedrich D. (2018). A systematic review on models of care effectiveness and barriers to Hepatitis C treatment in prison settings in the EU/EEA. J. Viral Hep..

[27] Fiore V., De Matteis G., Pontali E., De Vito A., Panese S., Geremia N., Maida I., Artioli S., Starnini G., Madeddu G. (2022). Quick diagnosis, staging, and treatment of HCV infection among people living in prison: Opinion expert panel. Front. Public Health.

[28] Beckwith C.G., Kurth A.E., Bazerman L., Solomon L., Patry E., Rich J.D., Kuo I. (2015). Survey of US Correctional Institutions for Routine HCV Testing. Am. J. Public Health.

